# The impact of disease severity adjustment on hospital standardised mortality ratios: Results from a service-wide analysis of ischaemic stroke admissions using linked pre-hospital, admissions and mortality data

**DOI:** 10.1371/journal.pone.0216325

**Published:** 2019-05-21

**Authors:** Melina Gattellari, Chris Goumas, Bin Jalaludin, John Worthington

**Affiliations:** 1 Heart and Brain Collaboration, Ingham Institute for Applied Medical Research, Liverpool, Sydney, New South Wales, Australia; 2 Institute of Clinical Neurosciences, Royal Prince Alfred Hospital, Camperdown, Sydney, New South Wales, Australia; 3 Population Health Intelligence, Healthy People and Places Unit; South Western Sydney Local Health District, Liverpool, Sydney, New South Wales, Australia; 4 School of Public Health, The University of New South Wales, Kensington, Sydney, New South Wales, Australia; 5 South Western Sydney Clinical School, The University of New South Wales, Liverpool, Sydney, New South Wales, Australia; University of Malta Faculty of Health Sciences, MALTA

## Abstract

**Background:**

Administrative data are used to examine variation in thirty-day mortality across health services in several jurisdictions. Hospital performance measurement may be error-prone as information about disease severity is not typically available in routinely collected data to incorporate into case-mix adjusted analyses. Using ischaemic stroke as a case study, we tested the extent to which accounting for disease severity impacts on hospital performance assessment.

**Methods:**

We linked all recorded ischaemic stroke admissions between July, 2011 and June, 2014 to death registrations and a measure of stroke severity obtained at first point of patient contact with health services, across New South Wales, Australia’s largest health service jurisdiction. Thirty-day hospital standardised mortality ratios were adjusted for either comorbidities, as is typically done, or for both comorbidities and stroke severity. The impact of stroke severity adjustment on mortality ratios was determined using 95% and 99% control limits applied to funnel plots and by calculating the change in rank order of hospital risk adjusted mortality rates.

**Results:**

The performance of the stroke severity adjusted model was superior to incorporating comorbidity burden alone (c-statistic = 0.82 versus 0.75; N = 17,700 patients, 176 hospitals). Concordance in outlier classification was 89% and 97% when applying 95% or 99% control limits to funnel plots, respectively. The sensitivity rates of outlier detection using comorbidity adjustment compared with gold-standard severity and comorbidity adjustment was 74% and 83% with 95% and 99% control limits, respectively. Corresponding positive predictive values were 74% and 91%. Hospital rank order of risk adjusted mortality rates shifted between 0 to 22 places with severity adjustment (Median = 4.0, Inter-quartile Range = 2–7).

**Conclusions:**

Rankings of mortality rates varied widely depending on whether stroke severity was taken into account. Funnel plots yielded largely concordant results irrespective of severity adjustment and may be sufficiently accurate as a screening tool for assessing hospital performance.

## Introduction

Administrative data are widely used to examine variation in thirty-day mortality across health services in several international jurisdictions [1–6]. These analyses flag outlier hospitals associated with higher or lower than expected mortality, implying that variation in death rates are attributable in part to quality of care. As administrative data do not typically incorporate standardised measures of disease severity, a persistent criticism is that hospital performance measures do not adequately account for differences in case-mix between hospitals, risking misclassification of “performance outliers”.

It is widely assumed that severity adjustment is necessary for credible examination of inter-hospital variability. However, evidence that this improves the accuracy of hospital profiling is scant. Moreover, different methods are used to assess hospital performance, including statistical control charts and “league tables” that rank order hospitals [1–6]. It is unclear whether the impact of disease severity adjustment depends on how hospital performance is determined.

In stroke medicine, adjusting for disease severity improves the prediction of risk-adjusted models [1,7–9] inviting the conclusion that severity adjustment is crucial for valid hospital profiling. However, as risk adjustment is primarily concerned with reducing confounding arguably the emphasis should be on determining the impact of severity adjustment on hospital outlier status. Only two studies have addressed the impact of ischaemic stroke severity adjustment on hospital outlier status using a gold-standard measure of severity, the National Institutes of Health Stroke Scale (NIHSS) [7,8]. Results were conflicting and limitations included substantial missing stroke severity data (>55%) [8], and analyses involving select patients and hospitals [7,8], such as those participating in voluntary quality improvement programs with questionable representation [8].

Obtaining unbiased measures of stroke severity is challenging and measurement will be subject to error if severity is not assessed as close as possible to stroke onset and if documentation is incomplete [1,8,10,11]. For example, severity measured several hours after admission may capture deterioration or improvement, which partly reflects quality of care particularly in the context of time-critical interventions. Severity assessment also may be error-prone if retrospectively ascertained, inferred from clinical notes and/or recorded unblinded to clinical progression, in-patient mortality, or discharge status. One recent study noted that NIHSS scores measuring ischaemic stroke severity were preferentially documented in patients receiving thrombolysis or with severe stroke [11]. The same study and another noted documentation significantly varied between hospitals [10,11]. Therefore, inconsistencies in severity documentation rather than severity per se, may in part underpin inter-hospital variation in mortality. Missing stroke severity data may bias hospital mortality estimates towards greater risk because deaths cannot be attributed to prognostic indicators while more diligent recording allows hospitals to ascribe fatalities to severe stroke. Ideally, risk-adjusted models incorporating stroke severity indicators that are standardised, universally available, reliably assessed in real-time at first point of contact with health services and recorded blinded to processes of care and outcomes are needed [1]. However, severity indicators meeting these criteria are yet to be widely implemented.

We sought to address these two gaps in the current literature. We first developed a risk adjusted model incorporating a universal, real-time stroke severity measure ascertained prospectively and independently of hospital staff managing patients and blinded to processes of care and patient outcomes. Applying this model, we then determined the accuracy of comorbidity-adjusted hospital performance measures against gold-standard severity adjustment, assessing the extent to which comorbidity adjustment produces valid results for hospital performance evaluation. Further, we compared two common approaches to assess hospital performance, specifically statistical control charts and league tables to test whether the accuracy of comorbidity adjustment depends on how performance is determined.

## Material and methods

Analyses presented here are part of the Home to Outcomes Study (H20), which aims to assess stroke health service management and epidemiology across New South Wales (NSW), Australia’s largest jurisdiction (population ~7.7 million). Pre-hospital vital signs data informed stroke severity at onset and were linked to routinely collected hospital admissions and emergency department (ED) data (S1 Appendix). As described elsewhere [12], data linkage was carried out by a government provider of linkage services using ethically approved gold-standard and privacy preserving protocols. Deterministic and probabilistic linkages were undertaken to enable de-identified patient level-analysis. Variables utilised for linkage included patient identifiers and dates of health service delivery (for example, dates of admission and discharge, transport dates). Automated algorithms for linkage, supplemented by manual quality assurance checks ensured that the false-positive linkage rate for the project was less than 5 per 1,000.

### Ethics statement

The NSW Population and Health Services Ethics Committee approved the study (HREC/14/CIPHS/17) allowing a waiver of consent as the study utilised routinely collected data from which direct patient identifiers were removed.

### Patient selection

We identified ischaemic stroke admissions from all public hospitals in NSW from July, 2011 to June, 2014 using the Admitted Patient Data Collection (APDC), a census of all NSW hospitalisations. The APDC records the main reason for the admission (principal diagnosis) and up to 49 comorbid diagnoses according to the International Classifications of Diseases, Version 10, Australian Modification (ICD-10AM) [13]. We selected all admissions with a principal diagnosis of ischaemic stroke in patients aged 15 years or older using ICD-10AM codes 163 and I64 as elsewhere [13–16]. Acute strokes recorded in secondary positions were included upon manual review with agreement amongst the authors if the principal diagnosis was consistent with a stroke presentation unless flagged as an in-hospital event or as a post-procedural stroke. We excluded strokes recorded more than three days after admission as presumed in-hospital events to avoid ascribing a severity score recorded distally from the stroke diagnosis and for a condition unrelated to stroke. Cases with concomitant codes for primary or metastatic cerebral neoplasms and severe head injury or trauma were ineligible complying with accepted “gold standard” case selection for stroke [17].

Other exclusion criteria were applied to reduce misclassification of case selection. We excluded patients with stroke diagnoses revised to other diagnoses upon transfer to another hospital by the end of the next day after presentation [2] as such cases reduce the accuracy of cohort ascertainment [18] and their retention may systematically bias HSMR estimates. In cases transferred soon after presentation, a discharge diagnosis of stroke may indicate a provisional diagnosis while in non-transferred cases working diagnoses will instead be discarded in favour of the established reason for the hospital admission. In hospitals with relatively low transfer rates, final diagnoses will more likely represent stroke cases, while a discharge diagnosis of stroke may represent both confirmed and provisional stroke diagnoses in hospitals more likely to transfer patients, potentially leading to differential case ascertainment between hospitals with different transfer rates. Cases discharged home alive within 48 hours were also excluded as potential misclassified strokes, consistent with our *a-priori* case selection as reported elsewhere [12], and with approaches to improve the specificity of case selection [19–21]. While early discharges may include very mild strokes, symptom resolution allowing discharge within a short time was considered to be more likely to indicate either transient ischaemic attacks or provisional stroke diagnoses given almost half (48.4%) were coded as non-specified strokes. We undertook a post-hoc sensitivity analysis to determine the effect of these two exclusion criteria on study findings.

Non-NSW residents and those discharged outside the state were also excluded to improve the accuracy of 30-day mortality ascertainment as deaths occurring outside our jurisdiction are not usually recorded in the NSW death register. We attributed care to the hospital of first presentation akin to an intention-to-treat principle^,^ as the decision to retain or transfer patients has ramifications for patient outcomes [2]. This was considered to allow fairer assessment for centres which have implemented pathways for inter-hospital transfers as opposed to others which have not structured services to enable patient access to better resourced facilities. For patients with multiple hospitalisations, we selected the first admission (“index admission) for analysis in keeping with other approaches [3].

### Outcome measure

Thirty-day mortality was derived from linking admissions to the NSW Registry of Birth, Deaths and Marriages which records all deaths occurring in NSW.

### Risk adjusted models

We defined a base model comprising year of admission, sex, stroke history, a measure of socioeconomic status derived from geographical location of patient residence [22] and age. We added atrial fibrillation (ICD-10 code I48) and a modified, validated version of the Charlson Comorbidity Index [23,24] to produce comorbidity risk-adjusted estimates (Model 1, comorbidity adjusted). To reduce misspecification, we modelled linear and quadratic terms for age and summed empirically derived weights for individual Charlson comorbidities. Stroke history (ICD-10-AM I60-I64, I62.9, I69.0-I69.4) and comorbidities were ascertained by interrogating linked admissions recorded during the stroke admission and the maximum available “look-back” period of ten years.

A second model incorporated a five-point measure of stroke severity (Model 2, severity and comorbidity adjusted) based on the Glasgow Coma Scale Score (GCS) [25] and arrival to hospital by private transport, both prognostic of ischaemic stroke mortality [1,26–28].

Data for patients accessing ambulance transport were linked to GCS scores assessed by paramedics at time of first contact with patients, as recorded in the Ambulance NSW electronic medical record (eMR) and Patient Health Care Record (PHCR) data-sets. GCS scores were complete in almost all eligible cases (99.4%, N = 79 missing cases) providing a universal measure of stroke severity recorded in real-time closest to onset for almost every recorded stroke patient accessing NSW ambulance services. Patients were categorised as fully conscious (GCS = 15), or as having Mild (GCS = 13–14); Moderate (GCS = 9–12) or Severe (GCS = 3–8) brain injury using pre-specified cut-offs [26,27]. As GCS scores were not recorded for patients arriving to hospital using private transport, a fifth category was created presuming milder strokes at onset than for patients requiring paramedical assistance. Our measurement of “mild” or “ambulant” stroke was therefore objectively and prospectively ascertained at presentation and recorded independent of clinicians managing patient care. Arrival to hospital via ambulance or private transport was identified using linkage to ambulance data.

We evaluated model goodness of fit using the Aitake Information Criteria (AIC). Calibration was determined using the Brier score and Nagelkerke generalised r^2^, while the c-statistic assessed discrimination. Higher c-statistics and Nagelkerke r^2^ values and lower Brier scores and AIC values indicated better model performance. C-statistic values, ranging from 0 to 1 indicate the probability that a randomly selected patient dying within 30-days had a higher predicted probability of dying than a patient surviving to Day 30 [3]. Values greater than 0.70 or 0.80 indicate a reasonable or strong model, respectively, while a c-statistic of 0.50 indicates categorisation of vital status is no better than chance [3]. Performance was validated against an external data-set applying the same selection criteria used to derive data for the main study period. The external data-set comprised three years of linked administrative data recording information about ischaemic stroke patients from July 1, 2008 to June 30, 2011.

### Hospital profiling

Hospital risk-adjusted standardised mortality ratios (HSMRs) were calculated by dividing the observed by the expected number of deaths obtained from logistic regression parameter estimates for each model tested [2,29–31]. Hospital was fitted as a random intercept using PROC GLIMMIX (SAS 9.4, SAS institute Inc, Cary, USA) accounting for patient data clustered within hospital. This approach compares the hospital-specific mortality rate to the expected risk of death for patients treated in a hospital with “average mortality” [2,29–31]. All admissions contributed to risk adjustment, while HSMRs were computed for hospitals with at least one observed and one expected death thereby removing highly unreliable HSMRs from analysis [2,32]. HSMRs were plotted using statistical control charts also known as funnel plots. This approach places a higher threshold for meeting outlier status for hospitals with a relatively small caseload compared to centres with higher throughput.

#### Impact of stroke severity adjustment: Standard models

We identified outlier hospitals with higher or lower than expected mortality using funnel plots generated by plotting HSMRs against the expected number of deaths, with control limits constructed using a gamma-distribution applied to the number of observed deaths. Hospitals falling outside 95% and 99% control limits were designated outliers representing liberal and conservative criteria for an “alert” signal selected to inform quality improvement activities [2,3,33,34]. These respective control limits indicated HSMRs around 2 and 2.5 standard deviations above or below the expected mortality rate. Results applying 99.8% control limits indicating ± 3 standard deviations around the expected mortality rate favoured to minimise false-positive results, are reported as sensitivity analyses.

HSMR outliers derived from Model 2 (severity and comorbidity adjusted) were the gold-standard against which Model 1 derived outliers (comorbidity adjusted) were compared. Positive Predictive Values (PPV), sensitivity and false-positive (FP) and false-negative (FN) rates were computed. Concordance of outlier classification was calculated using percentage agreement and the Kappa statistic [35] (Medcalc version 17.9.7, Medcalc Software, Ostend, Belgium).

Risk-adjusted mortality rates (RAMRs) were obtained by multiplying the HSMRs by the overall crude mortality rate. RAMRs derived from Model 1 and Model 2 were rank ordered, reflecting the commonly used “league table” approach in public reporting [1]. Agreement between RAMRs was assessed using Bland-Altman Plots [36], graphing differences in rank order against the mean rank derived from comorbidity risk adjusted models with and without severity adjustment. 95% upper and lower bounds of agreement representing two standard deviations away from the mean rank difference were superimposed on the plot and the dispersion of data points around the mean difference was visually examined. Greater agreement is suggested by narrower 95% bounds of agreement around the mean difference.

Funnel plot analyses included all eligible hospitals while analyses of rank order excluded hospitals with fewer than 25 strokes to minimise random error in RAMRs [8]. Correlations were calculated using the Spearman rho statistic.

#### Impact of stroke severity adjustment: Enhanced risk adjustment models

We hypothesised that additional prognostic information will improve both model performance and the accuracy of HSMRs, particularly for Model 1 which adjusted for comorbidity burden alone. We added mode of hospital arrival (ambulance versus private transport), and the most urgent triage level mandating immediate treatment upon ED presentation, as severity variables prospectively assessed in real-time. A GCS score less than 9 is one indicative criterion qualifying for immediate treatment in ED [37]. These real-time prognostic indicators are not widely available [1–6]. In our jurisdiction these are reliably ascertained via linkage to ED and ambulance data. We compared model performance and outlier classification of this enhanced comorbidity adjusted model (Model l) with the severity and comorbidity adjusted model (Model 2) enhanced by incorporating urgent triage.

## Results

### Patient and hospital Cohort

The crude 30-day mortality rate was 14.8% (N = 2,613 deaths) based on data from 17,700 eligible ischaemic stroke patients treated in 176 hospitals (S1 Appendix). Stroke severity as measured using the GCS was strongly associated with 30-day mortality (Table 1). Of the 5,164 (29.2%) ischaemic stroke patients arriving to hospital using their own transport, 276 (5.3%) died within 30-days, while 8.8% with GCS scores of 15 died within 30-days. Almost one-fifth of patients with a GCS score of 13–15 died within 30-days, increasing to 38.5% and 65.4% of those with initial GCS scores of 9–12 and 3–8, respectively. Patient characteristics by vital status are shown in S2 Appendix.

**Table 1:** Mortality by stroke severity.

Stroke severity measure	N Died (%, 95% CI)	Total	Adjusted Odds Ratio*
Ambulant**	276 (5.3;4.8–6.0)	5,164	Ref
GCS = 15	655 (8.8;8.2–9.5)	7,406	1.27 (1.09–1.47)
GCS = 13–14 (Mild)	434 (17.8; 16.3–19.1)	2,438	2.13 (1.80–2.53)
GCS = 9–12 (Moderate)	733 (38.5;36.3–40.7)	1,905	6.38 (5.43–7.50)
GCS = 3–8 (Severe)	515 (65.4;62.1–68.9)	787	20.52 (16.80–25.0)

*Adjusted for Age, age^2^, sex, year of admission, prior stroke, socio-economic status, Charlson comorbidities, Atrial Fibrillation

**Patient arrived to hospital using their own transport.

### Measuring the impact of stroke severity (Standard models)

#### Model performance

Adjusting for stroke severity in addition to comorbidity burden (Model 2) versus comorbidity adjustment alone (Model 1) produced superior model performance statistics. The c-statistic for the model adjusting for comorbidities only was 0.75, compared to 0.82 when adding stroke severity (Table 2). Performance statistics were replicated when validated against an external data-set (S3 Appendix).

**Table 2:** Model performance statistics (Standard Models).

Model	Akaike Information Criterion	Nagelkerke r^2^	c-statistic (95% CI)	Brier Score
Base Model*	13,460	0.13	0.72 (0.71–0.73)	0.12
Model 1: Base+Comorbidities**	13,048	0.17	0.75 (0.74–0.76)	0.11
Model 2: Base+Comorbidities+Stroke Severity	11,487	0.31	0.82 (0.82–0.83)	0.10

*Age, age^2^ (quadratic term), sex, year of admission, prior stroke and measure of socio-economic status.

**Charlson comorbidities, Atrial Fibrillation.

HSMRs for 30-day mortality were calculated for 114 hospitals treating 17,451 patients with at least one observed and one expected death. Classification of HSMRs as within or outside the expected range was concordant between the two models for 102 (89%) and 111 (97%) hospitals when applying 95% or 99% control limits, respectively. Kappa statistics demonstrated “substantial” or “almost” perfect agreement [35] in outlier classification between Model 1 (comorbidity adjusted) and Model 2 (severity and comorbidity adjusted) (Table 3).

**Table 3:** Number of outliers and inliers using comorbidity adjusted (Model 1) funnel plot results compared against “gold standard” severity and comorbidity adjusted (Model 2) funnel plot results (Standard Models).

Gold Standard Risk Adjustment (Model 2) N = 114 Hospitals				
	**95% Control Limits**		**99% Control Limits**	
***Comorbidity adjustment (Model 1)***	***Number of outliers***	***Number of inliers***	***Number of outliers***	***Number of inliers***
Number of Outliers	17	6	10	1
Number of Inliers	6	85	2	101
**Accuracy Metric**	**95% Control Limits**		**99% Control Limits**	
Percent agreement*	(17+85)/114 = 89%		(10+101)/114 = 97%	
Kappa (95% CI)	0.67 (0.50–0.84)		0.85 (0.69–1.00)	
Sensitivity (95% CI)^†^	17/(17+6) = 74% (52%-90%)		10/(10+2) = 83% (52%-98%)	
PPV (95%CI)^††^	17/(17+6) = 74% (56%-86%)		10/(10+1) = 91% (58%-98%)	
False Positive Rate^‡^	6/(6+85) = 7%		1/(1+101) = 1%	
False Negative Rate^‡‡^	6/(6+17) = 26%		2/(2+10) = 17%	

*Percentage of hospitals with concordant classifications between comorbidity (Model 1) and gold-standard adjustment (that is, comorbidity and severity adjustment, Model 2).

†Proportion of true outliers according to gold-standard comorbidity and severity risk adjustment (Model 2) detected as outliers according comorbidity adjustment alone (Model 1).

††Proportion of outliers detected by comorbidity risk adjusted modelling (Model 1) that are true outliers according to gold-standard severity and comorbidity adjustment (Model 2).

‡Number of hospitals falsely detected as outliers using comorbidity risk adjustment alone (Model 1) divided by the number of “inlier” hospitals according to gold-standard risk adjustment (Model 2).

‡‡Number of hospitals missed as “true outliers” using comorbidity risk adjustment alone (Model 1) divided by the number of “true outlier” hospitals according to gold-standard risk adjustment (Model 2).

#### Analysis of funnel plots: 95% control limit outliers

**Fig 1:**
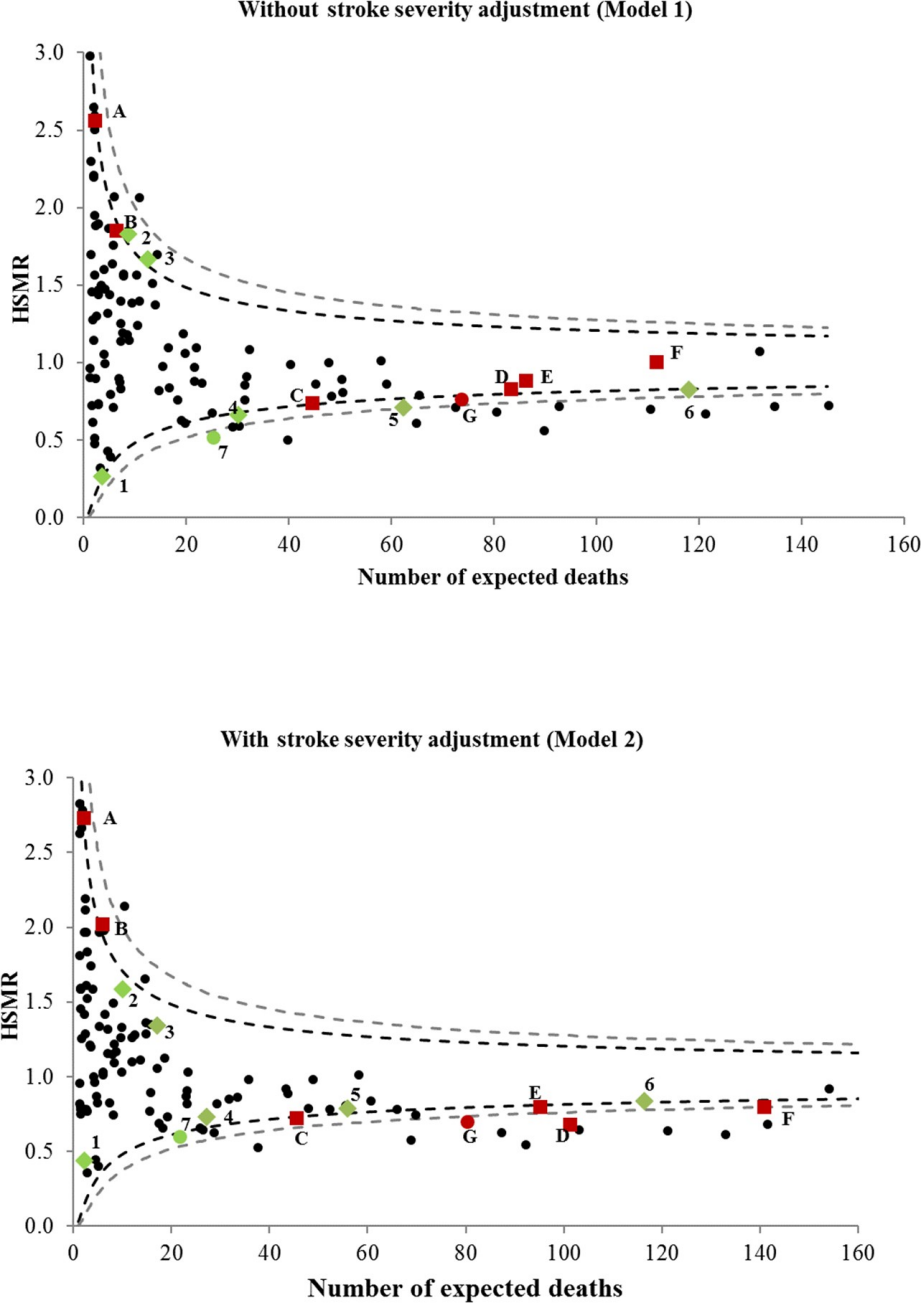
Change in outlier status of comorbidity adjusted HSMRs without and with severity adjustment. --- 95% Control Limits--- 99% Control Limits Stroke severity adjustment changes health service from an outlier to a non-outlier designated with a green diamond Stroke severity adjustment changes health service from a non-outlier to an outlier designated with a red square Alert signal “downgraded” from an 99% to 95% control limit outlier with stroke severity adjustment designated with a green circle. Alert signal “upgraded” from an 95% to 99% control limit outlier with stroke severity adjustment designated with a red circle.

Twenty-three comorbidity adjusted HSMRs (Model 1) were identified as outlier services. Five were flagged as having higher than expected mortality. Seventeen, including three with higher than expected mortality, were “true” outliers with gold-standard severity and comorbidity adjustment (Model 2) (PPV = 74%, FP = 7%) (Table 3; Fig 1).

Gold-standard stroke severity and comorbidity risk adjustment (Model 2) yielded 23 “true” HSMR outliers. Seventeen of these were detected with comorbidity adjustment alone (Model 1, Sensitivity = 74% and FN = 26%, Table 3). Five outliers had higher than expected mortality identified with gold-standard severity and comorbidity adjustment (Model 2). Three of these were flagged as outliers with only comorbidity adjustment (Model 1) (Table 3; Fig 1).

#### Analysis of funnel plots: 99% control limit outliers

Eleven comorbidity adjusted HSMRs were detected as outliers (Model 1). Of these, ten were “true” outliers with severity and comorbidity adjustment (Model 2) (PPV = 91%, FP = 1%, Table 3). There were twelve “true” outlying severity adjusted HSMRs (Model 2). Ten of these were detected using comorbidity adjustment alone (Model 1) (Sensitivity = 83%, FN = 17%) (Table 3; Fig 1).

#### Analysis of funnel plots: 99.8% control limits (sensitivity analysis)

When applying 99.8% control limits, all seven outliers detected using comorbidity adjustment alone were also detected as “true” outliers with both comorbidity and severity adjustment (PPV = 100%). Comorbidity adjustment detected seven out of eleven “true” outliers (Sensitivity 64%, Kappa = 0.76, 95% CI = 0.53–0.98, 97% concordance).

#### Rank order of RAMRs

For the 78 hospitals with at least 25 cases, 30-day crude mortality rates ranged from 7.8% to 41.4% (Median 15.6%, IQR = 12.8%-23.2%). Rates ranged from 5.8% to 30.6% (Median 13.2, IQR = 10.9–17.7%) when adjusted for comorbidity burden (Model 1) and 6.7% to 31.7% (Median 12.9%, IQR = 10.8–17.1) when adjusted for both stroke severity and comorbidity burden (Model 2).

**Fig 2:**
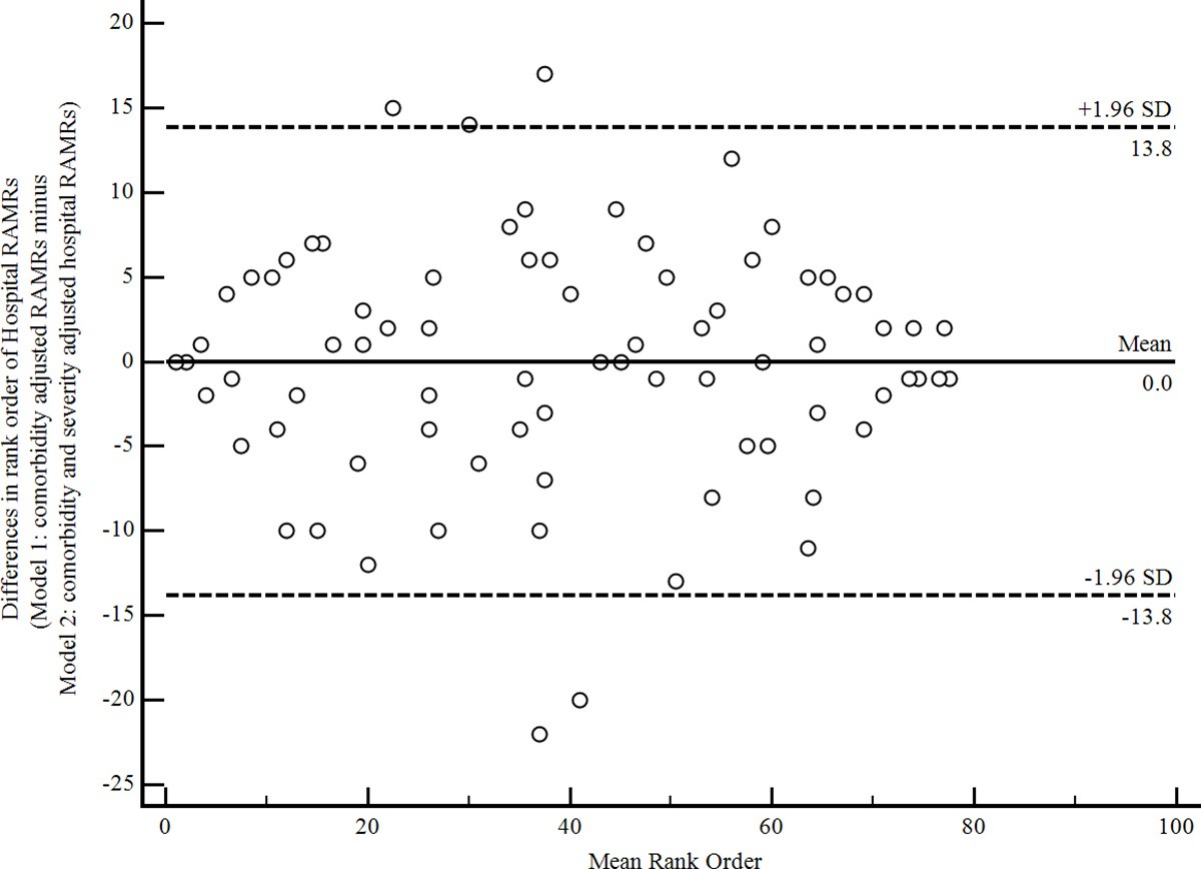
Bland-Altman plot displaying differences in rank order comorbidity adjusted RAMRs with and without severity adjustment.

The lower and upper bounds of agreement showed that 95% of rank order differences ranged from -13.8 to 13.8 with wide dispersion around the mean rank difference (Fig 2). The absolute change in hospital rank order of RAMRs obtained from Model 1 and Model 2 ranged from 0 to 22 (Median = 4.0, IQR = 2–7). The median absolute difference in RAMRs was 1.0% (IQR = 0.51%;2.0%). The change in hospital rank order correlated moderately with absolute differences in RAMRs (rho = 0.39; 95% CI = 0.18–0.56; p = 0.0004). Relatively small changes in rank order were associated with large differences in RAMRs and vice versa. For example, the greatest change in rank of 22 places corresponded to a 3.1% difference in RAMRs and a difference of one expected death over the three-year study period.

### Measuring the impact of stroke severity adjustment (Enhanced models)

#### Model performance

The c-statistic for Model 1 (comorbidity adjusted) improved from 0.75 to 0.80 when enhanced by adding mode of arrival (ambulance versus private transport) and the most urgent ED triage category. In comparison, discrimination improved marginally for Model 2 (severity and comorbidity adjusted) when enhanced by adding triage category (c-statistic = 0.83 versus 0.82) although this enhanced model was the best performing of all those tested (Tables 2 and 4). These enhanced models were well-validated (S3 Appendix).

**Table 4:** Model performance statistics (enhanced models).

Model	Akaike Information Criterion	Nagelkerke r^2^	c-statistic (95% CI)	Brier Score
Base	13,460	0.13	0.72 (0.71–0.73)	0.12
Model 1: Base*+Comorbidities**+Arrival by ambulance versus private transport+Urgent triage category	12,145	0.25	0.80 (0.79–0.81)	0.11
Model 2: Base*+Comorbidities**+Stroke severity†+Urgent triage category	11,334	0.32	0.83 (0.82–0.84)	0.10

*Age, age^2^ (quadratic term), sex, year of admission, prior stroke and measure of socio-economic status.

**Charlson comorbidities+Atrial Fibrillation.

†Arrival by private transport is included in the stroke severity measure.

#### Analysis of funnel plots

When examining funnel plots, concordance in hospital outlier classification between these two enhanced models was almost perfect (~95% using 95% and 99% control limits, respectively) (Table 5; S4 Appendix). HSMRs were highly correlated (rho = 0.98, 95% CI = 0.97 to 0.99) (p<0.0001).

**Table 5:** Number of outliers and inliers using comorbidity adjusted (Model 1) funnel plot results compared against “gold standard” severity and comorbidity adjusted (Model 2) funnel plot results (Enhanced Models).

Gold Standard Risk Adjustment (Model 2) N = 113 Hospitals				
	**95% Control Limits**		**99% Control Limits**	
**Comorbidity adjustment (Model 1)**	Number of outliers	Number of inliers	Number of outliers	Number of inliers
Number of Outliers	24	6	13	4
Number of Inliers	0	83	0	96
**Accuracy Metric**	**95% Control Limits**		**99% Control Limits**	
Percent agreement*	(24+83)/113 = 95%		(13+96)/113 = 96%	
Kappa (95% CI)	0.85 (0.74–0.97)		0.85 (0.70–0.99)	
Sensitivity (95% CI)^†^	24/24 = 100% (86%-100%)		13/13 = 100% (75%-100%)	
PPV (95%CI)^††^	24/(24+6) = 80% (65%-90%)		13/(13+4) = 76% (55%-89%)	
False Positive Rate^‡^	6/(6+83) = 7%		4/(4+96) = 4%	
False Negative Rate^‡‡^	0/24 = 0%		0/13 = 0%	

*Percentage of hospitals with concordant classifications between comorbidity (Model 1) and gold-standard adjustment (that is, comorbidity and severity adjustment, Model 2).

†Proportion of true outliers according to gold-standard comorbidity and severity risk adjustment (Model 2) detected as outliers according comorbidity adjustment alone (Model 1).

††Proportion of outliers detected by comorbidity risk adjusted modelling (Model 1) that are true outliers according to gold-standard severity and comorbidity adjustment (Model 2).

‡Number of hospitals falsely detected as outliers using comorbidity risk adjustment alone (Model 1) divided by the number of “inlier” hospitals according to gold-standard risk adjustment (Model 2).

‡‡Number of hospitals missed as “true outliers” using comorbidity risk adjustment alone (Model 1) divided by the number of “true outlier” hospitals according to gold-standard risk adjustment (Model 2).

Comorbidity adjusted HSMRs for 30 hospitals were outside 95% control limits. Twenty-four of these were “true” outliers with enhanced “gold-standard” stroke severity and comorbidity adjustment (Model 2) (PPV = 80%) (S4 Appendix).

When 99% control limits were applied, 17 outliers were identified using enhanced comorbidity adjustment with 13 signalling as “true” outliers with enhanced severity adjustment (PPV = 76%). The four “false-positives” were, however, identified as “true” outliers using 95% control limits. Enhanced comorbidity adjustment detected all gold-standard enhanced severity and comorbidity adjusted HSMRs lying outside 95% (N = 24) or 99% (N = 13) control limits (Sensitivity = 100%) (Table 3).

Eleven outliers using comorbidity adjustment alone were identified using 99.8% control limits all of which were also severity and comorbidity adjusted outliers. Similarly, eleven out of 12 “true” outliers using severity and comorbidity adjustment were identified using comorbidity adjustment only (Concordance = 99%; PPV = 100%, Sensitivity 92%, Kappa = 0.95, = = 95% CI = 0.86–1.00).

#### Rank order of hospital RAMRs

Hospital RAMRs ranged from 6.3% to 34.9% when adjusted for comorbidities alone and 7.0% to 31.5% when adjusted for both stroke severity and comorbidities. The median change in absolute differences in the hospital rankings produced from the two enhanced models was three (IQR = 1;5) and ranged from 0 to 17. Bland-Altman plots revealed upper and lower 95% confidence limits of agreement of 9.8 ranks.

Our post-hoc sensitivity analysis including presumed misclassified cases of stroke yielded similar results to the original analysis (S5 Appendix).

#### Stroke unit care and hospital performance

We undertook a post-hoc examination to determine whether the 31 hospitals offering stroke unit care in NSW, 19 of which had 24/7 acute thrombolysis capacity, would have lower than expected mortality. The median SMR for hospitals without stroke units was 1.19 (Interquartile range = 0.85 to 1.61), indicating the median mortality rate was 20% higher than expected in hospitals without stroke units. In contrast, the median SMR for hospitals offering stroke units with or without 24/7 thrombolysis capacity indicated the median mortality rate was 39% and 19% lower than expected, respectively (Median 0.61, Interquartile range = 0.60 to 0.78 and Median 0.81, Interquartile range = 0.77 to 0.83, respectively) (Kruskall-Wallis test = 33.2, df = 2, p<0.001).

Twenty hospitals had lower than expected mortality using HSMRs derived from the best performing enhanced “gold-standard” severity and comorbidity risk-adjusted model (Model 2) using 95% control limits. These hospitals included 17 offering stroke units, twelve of which also had 24/7 thrombolysis capability. None of the four hospitals with higher than expected mortality offered stroke unit care. When analyses were restricted to the peer-group of 31 hospitals with stroke unit facilities, six signalled as outliers with 95% control limits applied to HSMRs derived from the enhanced “gold-standard” model. All six were identified with comorbidity adjustment alone with no additional outliers flagged. Therefore, complete concordance between results adjusting for comorbidities with and without severity adjustment was achieved.

## Discussion

### Main findings

We found that the impact of stroke severity adjustment on hospital performance assessment depended on how it was assessed. HSMRs calculated using comorbidity burden with or without stroke severity adjustment often generated dramatic differences in hospital rank and the extent of disagreement largely precluded interchanging rank order. In contrast, identifying hospital outliers using funnel plots produced largely concordant results whether or not the severity measure was incorporated into the risk adjusted model. This was particularly the case when broader control limits were applied to signal outlier status or when model performance was enhanced with additional prognostic indicators, specifically ED triage information.

Concordance of outlier classification was at least 89% irrespective of which “alert” limits were applied. Varying control limits altered the sensitivity and specificity of the outlier signal. Both the sensitivity and PPV for the standard comorbidity adjusted model were 74% when using 95% control limits, increasing to 83% and 91%, respectively, when 99% control limits were applied. Applying 99.8% control limits increased the PPV of comorbidity adjustment results further.

By comparison, there was limited agreement between the rank order of hospital specific RAMRs with and without accounting for stroke severity. However, changes in rank order were largely inconsequential. For 50% of hospitals, differences in RAMRs generated from the two models were less than 1%. League tables have been criticised for overstating spurious differences between hospitals [38] and for being excessively susceptible to random variation [39]. These results show that rank order methods also exaggerate differences between risk adjusted models incorporating different prognostic indicators. There are two main implications of these findings. First, rank ordering hospitals is vulnerable to error when risk adjustment does not account for stroke severity. Second, using statistical control charts to measure hospital performance appears to limit misclassification of outliers when identified using comorbidity adjustment alone.

Misclassification of hospital outliers derived using comorbidity adjustment alone was minimised when prognostic indicators were added to enhance risk adjustment. Including ED triage data and mode of arrival improved model performance, increased the sensitivity of outlier identification and also reduced rank order differences. The sensitivity of outlier ascertainment with this enhanced comorbidity adjusted model was 100%, irrespective of whether 95% or 99% control limits were applied to funnel plots. There were no false negatives while false positives appeared unaffected with stringent limits. Whether comorbidity risk-adjusted models are sufficiently accurate cannot be completely resolved using statistical methods and inevitably involves a subjective judgement. When analyses are applied to screen hospitals for further review, a higher proportion of false-positives may be tolerated to maximise sensitivity and reduce false negatives. Data used to justify financial incentives or disincentives will likely demand higher specificity and therefore more stringent control limits may be warranted [31,33,34]. On the other hand, a patient-centred approach justifies maximising sensitivity as patients may eschew methods that deliberately reduce the risk of false positives and potentially increase the number of false negatives.

### Comparison with previous research

Stroke severity adjustment improved model performance when predicting mortality consistent with previous research [7–9]. However, whether stroke severity adjustment is necessary for valid outlier assessment is uncertain. Two previous studies measured hospital performance with and without ischaemic stroke severity adjustment using the NIHSS involving 782 [8] and 64 [7] hospitals. Another study of 28 hospitals measured ambulation upon admission as a proxy stroke severity measure without distinguishing ischaemic from haemorrhagic stroke or using nested models to systematically test the impact of severity adjustment [40].

Consistent with findings reported here, changes in rank order in two of the studies indicated substantial differences according to whether case-mix accounted for severity [8,40]. One of these studies used the NIHSS [8], we used a measure based on GCS scores and others measured “ability to walking upon admission” [40]. The existing evidence therefore suggests that ranking methods do not produce reliable results without stroke severity adjustment, and the evidence supports this to be the case irrespective of how severity is measured. Another study, however, found severity adjustment did not significantly alter hospital specific mortality rates, although results were inconclusive due to an overall low mortality rate unlikely to be representative (<5%) [7].

We found reduced misclassification of comorbidity adjusted HSMRs when analysed using funnel plots. In contrast, another study demonstrated inaccurate outlier classification when assessed using either a rank order method or an analysis of 95% credible limits of hospital effect estimates to identify mortality rates divergent from expected values [8]. Previous studies did not compare funnel plot results with different risk adjustment methods, so we cannot make direct comparisons between our results and those reported elsewhere. We acknowledge that sub-optimal severity adjustment may have driven our finding of minimal misclassification here and we cannot rule out that a more sensitive measure of severity may have produced different results.

Previous research has been limited due to a large amount of missing severity data with evidence of non-random capture [8], retrospective assessment of severity [7,8,40], incomplete case ascertainment and voluntary hospital participation [8,40]. These factors may underpin the differences in findings reported here and elsewhere. Variations in the way hospitals measure stroke severity rather than differences in case-mix may explain the confounding effect of stroke severity in hospital performance assessment seen in previous analyses [1]. Large amounts of missing data may have introduced bias particularly if the proportion of missing cases varied between hospitals, indicating differential bias in severity ascertainment. A recent publication has reported that missing severity data leads to inaccuracies in outlier classification, although residual bias remained, particularly for smaller hospitals [41]. Our severity measure is less prone to selective assessment with the advantage of maximising available cases for analysis (and therefore power), and minimizing differential ascertainment.

Selective severity assessment and non-standardised methods for assessing and reporting severity between hospitals will also bias hospital performance metrics. For example, there is evidence that severity recording varies between hospitals and is preferentially recorded in patients undergoing thrombolysis and those with severe stroke [10,11]. As robust measures of severity such as the NIHSS are best assessed by those with training and accreditation, assessors may differ in their reliability of assessment. Other sources of differential severity ascertainment include variability in the timing of assessment in relation to stroke onset. When severity is recorded prospectively in real-time or inferred retrospectively from medical notes, the quality and depth of information available for assessment is likely to differ. In hospitals offering thrombolysis the recorded NIHSS may be more likely to be prospectively recorded than hospitals not offering thrombolysis or with lower uptake. Severity may be preferentially recorded or be more accurately inferred in patients to facilitate and reflect end of life care, for those requiring rehabilitation, or to explain patient mortality. A lack of blinding to patient outcome may be particularly biasing. If the reasons for severity assessment differ across hospitals, then confounding is created by different hospitals assessing and reporting severity in different patients for different reasons. In our analysis, these potential sources of differential severity ascertainment are not present as hospital processes do not influence which patients receive severity assessment. The advantage of more sensitive severity assessment may be offset by these potential sources of bias when the NIHSS is used to risk-adjust hospital performance measures.

Previous studies included hospitals volunteering their participation in registries [8,40]. In contrast, our assessment is health system-wide, non-selective and includes rural and urban centres, facilities which do and do not offer stroke units and/or thrombolysis, and high and low volume hospitals, supporting the generalisability of our findings. While it is unclear how heterogeneity of hospitals affects case-mix adjustment and hospital profiling, we found evidence for the validity of comorbidity adjusted HSMRs whether analyses were system wide or when restricted to hospitals offering stroke units, although accuracy was greater with the latter. More research is required to examine the circumstances in which severity adjustment is more or less critical to hospital performance assessment. However, stroke severity may be more likely to confound the association between hospital and patient mortality in an analysis of hospitals volunteering their inclusion and with selective NIHSS assessment because stroke severity may differ between hospitals more widely than would be expected in a representative cohort with universal stroke severity assessment.

### Ascertaining stroke severity

We have demonstrated the feasibility and validity of a cost-effective approach for measuring stroke severity across a large health system which is *“captured among all patients and reliably recorded for all hospitals”*, [1] as recommended. Independent and standardised real-time assessment is invulnerable to biases that occur with retrospective ascertainment or severity scoring unblinded to patient outcome or processes of care.

To our knowledge, this is the first study assessing the impact of stroke severity adjustment on hospital profiling using real-time assessment of stroke severity at first point of patient contact with the health system, recorded blinded to processes of care and outcomes and ascertained by clinicians independent to those managing patients after admission. The universal coverage of severity assessment and mode of arrival to hospital–cost-effectively obtained–and of hospitals and patients within a large, representative and administratively defined state-wide jurisdiction are novel strengths of the analyses.

We acknowledge that the GCS, and our composite severity measure which assumes the mildest of strokes in patients arriving by private vehicles, public transport or walking, is not preferred over the NIHSS or other measures like it that are more sensitive in detecting stroke specific deficits. We use the GCS and our composite severity measure as a predictor of 30-day mortality and cannot be certain of the impact of scores such as the NIHSS in our analyses. To resolve this question, a definitive analysis would use the NIHSS measured in the same manner as the GCS here, that is, prospectively, blinded to patient outcome and ascertained by clinicians independent of those delivering the hospital care.

A validation of simpler measures of stroke severity has been advocated to facilitate wide-spread economical collection of severity information [1]. The GCS is a compelling candidate for inexpensive simplified stroke severity assessment given it is universally incorporated into emergency responder recordings of vital signs and integrated into criteria for the most urgent triage category upon emergency department presentation. Level of consciousness has been found to be a good proxy for the NIHSS for predicting 30-day ischaemic stroke mortality in a study validating prediction models in two separate validation cohorts [42]. The c-statistics for the model including the NIHSS were 0.86 for both cohorts restricted to ischaemic stroke cases. When the NIHSS was substituted with its four-point level of consciousness subscale, c-statistics were 0.81 and 0.86, respectively affirming good predictive accuracy. The broader range of scores in the GCS may allow better discrimination than that afforded by the 4-point consciousness NIHSS subscale. In another study of 1,217 patients, higher c-statistics were reported for the subset of dysphasic patients compared with the total cohort for models measuring mortality or residential care placement at 90-days, provided all GCS subscales were summed, allaying concerns that the GCS is not sufficiently sensitive to predict mortality in patients with focal neurological deficits [26].

In our study, model c-statistics, with and without enhancement (0.83 and 0.82, respectively), corresponded well with those derived from NIHSS adjusted models (for example, 0.79–0.82 [15], 0.82 [8] and 0.85 [28]) using 30-day mortality outcomes. As a prognostic indicator of 30-day mortality, our measure provided mortality rates comparable with those produced using a validated NIHSS categorisation [28]. For example, 5.3% of “ambulant” patients died within 30-days, compared with 4.2% for patients with low NIHSS scores (0–7) with 65.4% versus 54.5%, respectively, dying in severest GSC and NIHSS categories. In patients with GCS scores indicating moderate brain injury (scores between 9 and 12) 38.5% died, while 31.6% of patients with moderate to severe ischaemic stroke according to NIHSS classification died. While this should not imply that the GCS and the NIHSS are interchangeable, the comparison suggests convergent validity of these measures when assessing mortality following an ischaemic stroke.

The GCS has been shown to be a validated measure of mortality after ischaemic stroke [26,27] and arrival mode is also highly prognostic [eg 1,28]. Integrating ED triage information overcame limitations of using mode of arrival as triage criteria mandating immediate treatment include a GCS score of less than nine and other dire vital signs [37]. Therefore, our measures account for any relatively poor status in patients presenting without paramedical assistance and deterioration in the condition of patients delivered by ambulance with good initial vital signs.

Significant barriers exist to implementing the gold-standard NIHSS as a feasible universal measure. The NIHSS is frequently missing, retrospectively inferred from medical records and often recorded unblinded to patient deterioration or outcome in quality assurance registries [8,10,28]. Experts have called for the development of appropriate surrogate measures [1], which can be cost-effectively implemented on a system-wide scale, and our method does this. When severity adjusted HSMRs were also adjusted for urgent ED triage, 17 out of twenty hospitals with lower than expected 30-day mortality provided stroke unit care furnishing prima facie real-world validity for this model. As a group, these hospitals had lower than expected mortality compared with others while those offering thrombolysis had even lower rates. Access to stroke unit care should, when adjusted for severity indicators, translate to improved patient outcomes and our analysis provides evidence for effective knowledge translation. Further, the majority of hospitals with lower than expected mortality were higher volume providers, consistent with reports demonstrating lower mortality in higher caseload hospitals [43,44].

### Strengths and limitations

Potential limitations include the use of a severity measure that is not a gold-standard measure of stroke severity as discussed above. Coding inaccuracy is a potential limitation here although principal stroke diagnoses–the vast majority of admissions studied here (97.9%)–have been validated as 95% accurate within our jurisdiction [45,46]. Coding accuracy for cerebrovascular disease when validated against an “expert coder” was high in another Australian jurisdiction (Sensitivity = 89%, Positive Predictive Value = 93% and Kappa for inter-rater reliability = 0.91) [47]. Unspecified stroke cases (ICD-10 I64) were included in keeping with previous research [14–16]. The assumption these cases are predominately ischaemic is supported by validation studies that demonstrate incorporating unspecified strokes improves the sensitivity of ischaemic stroke ascertainment without compromising PPV [48].

Ambulance bypass may complicate hospital profiling analyses by altering case-mix. This may bias HSMRs against larger hospitals that have the capacity to manage more complex patients, making fair comparisons between hospitals challenging. However, we found that larger hospitals and those offering stroke units to which less well patients may be preferentially diverted, had lower than expected mortality providing limited evidence of bias against these hospitals with or without severity adjustment. In health systems where diversion is widespread, stratification of analyses by hospital characteristics is one approach to minimise confounding. However, system-wide analyses remains desirable and we have identified a feasible and cost-effective method for case-mix adjustment incorporating a measure of severity that is likely less biased than current methods.

The years under study predate evidence supporting mechanical thrombectomy. Rates of mechanical thrombectomy at the time were low and access was usually through inter-hospital transfers. Our analysis attributed care to the first presenting hospital on the grounds that decisions made at the first hospital have flow-on effects for patient outcomes with the gatekeeping of access to thrombectomy being just one of these decisions. This immunises against the biasing effect inter-hospital transfers have on hospital case-mix variability and system-wide linkage allows for reliable attribution of care. We note that the impact of endovascular clot retrieval on mortality was reported as neutral in a meta-analysis, although mortality has varied between trials [49]. If procedure coding can identify these cases, then the impact of these cases on hospital profiling can be researched.

Previous studies have each used different variables for comorbidity adjustment, although studies share common factors such as age, prior stroke, socioeconomic status and comorbidities relevant to stroke risk supporting the generalisability of findings, including our own [2–9,40]. We selected the Charlson Comorbidity Index, as this index is widely validated for 30-day mortality outcomes [50]. Our modelling strategy reduced misspecification and employed a long look-back period to minimise comorbidity misclassification which are strengths of the analysis, maximising accuracy. Analyses yielded similar model performance indicators suggesting generalisability of these measures.

We produced two sets of analyses, one using a standard risk adjustment model and another using an enhanced risk adjustment model. This had the effect of substantially improving model performance for the comorbidity adjusted model but only marginally so for the stroke severity adjusted model. Improved prediction also improved concordance between comorbidity and stroke severity adjusted models suggesting outlier misclassification is reduced with more optimal comorbidity adjustment.

Thirty-day mortality is widely favoured as a performance metric and therefore our results have direct application to analyses intended to inform health policy and practice. While mortality is easily quantified with high validity it is not the only outcome that matters in stroke. Low mortality rates may favour hospitals which more aggressively pursue life-sustaining interventions to prolong survival. We do not know the complex social and inter-personal communication that may have led to decisions influencing mortality rates and the capacity to measure the quality of decision-making in a systematic way is currently limited. Trade-offs between survival and disability are genuinely challenging for clinicians, patients and their families given evidence that prognostication is inexact [51]. The development of hospital performance metrics incorporating disability, quality of life outcomes and doctor-patient interactions is an area for future research.

We acknowledge that there are different methods for risk adjustment and calculating HSMRs. For example, risk adjustment may be performed using a fixed effects or random effects approach either with or without smoothing of observed counts, while HSMRs may be derived using direct or indirect standardisation [2,29–32,52,53]. No single method is universally preferred and every method has advantages and disadvantages. As opposed to using “smoothed” observed counts (ie shrinkage estimates), HSMRs calculated using observed deaths enable funnel plot analyses [2,29–31]. We were able to illustrate the validity or otherwise of two widely used approaches for hospital profiling and our comparisons derived from severity adjusted and unadjusted models were not confounded by the statistical approach. Two key considerations for valid modelling were met, namely accounting for inter-dependencies of outcome data within hospitals and minimising the instability of HSMRs for smaller hospitals.

## Conclusions

The validity of hospital performance measurement depends on the perceived credibility of analyses. Case-mix adjustment has traditionally accounted for comorbidity burden without including stroke severity measures or other prognostic indicators to enhance prediction [1–6]. Performance analysts have assumed that comorbidity adjustment serves as a valid proxy for disease severity despite significant disquiet about the accuracy of this approach [1].

Using a universal severity measure ascertained prospectively and in real-time, we reported that the impact of stroke severity adjustment on hospital rank order appeared impressive. However, differences in estimated mortality rates with and without stroke severity adjustment were often trivial. Our results therefore caution against using rank order methods, as hospital rankings appear greatly influenced by minor differences in RAMRs. Funnel plots were less vulnerable to differences in risk-adjustment and may be recommended to suggest further review in jurisdictions which either lack measures of severity or where there is evidence of biased and/or incomplete severity ascertainment.

We have also demonstrated the feasibility of linking routinely collected pre-hospital, hospital and mortality data to create a virtual stroke registry, cost-effectively integrating a measure of severity, to monitor outcomes and performance. Prehospital GCS combined with other variables from routinely collected data provided effective case mix adjustment for predicting 30-day mortality after stroke.

## Supporting information

S1 Appendix Data linkage and patient and hospital selection flow chart. (DOCX)

S2 Appendix Patient characteristics and comorbidities by vital status. (DOCX)

S3 Appendix Cross-validation of risk adjusted models. (PDF)

S4 Appendix Change in outlier status of comorbidity adjusted HSMRs with and without stroke severity adjustment (Enhanced models). (PDF)

S5 Appendix Sensitivity analysis of results including assumed “misclassified” stroke cases. (DOCX)
