# Supplementary material for: The impact of disease severity adjustment on hospital standardised mortality ratios: Results from a service-wide analysis of ischaemic stroke admissions using linked pre-hospital, admissions and mortality data
Source: PLoS One. 2019 May 21;14(5):e0216325. doi: 10.1371/journal.pone.0216325 (PMC6528964; doi:10.1371/journal.pone.0216325)
Supplement: S1 Appendix — (DOCX) [file pone.0216325.s001.docx]

**S1 Appendix: Linkage and patient and hospital selection flow chart.**

21,999 admissions identified

Exclusion criteria:

- Non-NSW residents: N=412
- Traumatic head injury : N=32**
- Misclassified stroke dx:: N=540
- Cerebral neoplasms: N=77**
- Early discharge home <48 hours: N=1,256
- Transferred outside NSW: N=399
- Post-procedural or in-hospital strokes: N=618

18,665 eligible admissions, of which 17,779 were index admissions (first admissions during study period)

N=79 arriving by ambulance excluded due to missing GCS scores.

Data from 17,700 patients treated in 176 hospitals linked to fact of death data registry (30-day mortality) contributed to risk adjustment†

Admitted Patient Data Collection (APDC)

Hospitalisations for Ischaemic Stroke

ICD-10AM (I63, I64)

N=17,779 Patients (15 years+)*

July, 2011 to June 30, 2014

N=17,451 patients from 114 Hospitals with 1+ observed and expected number of deaths

(Standard risk adjustment)

N=17,454 patients from 113 hospitals with 1+ observed and expected number of deaths (Enhanced risk adjustment)†

Pre-hospital Glasgow Coma Scale

N=12,526

Arrival to hospital using own transport

N=5,164

Linkage to up to ten years of previous hospitalisations (from July 1, 2001) to ascertain stroke history and comorbidities

Linkage to Pre-hospital and Emergency Department Data Collection (ED Data)

*The false-positive linkage rate was estimated to be no more than 5 per 1,000 records. **Cerebral neoplasms, including metastases ICD-10AM codes: ‘C71','C70.0','C70.9','C79.3','D33.0','D33.1','D33.2','D33.3','D33.9'.

Traumatic Head Injury ICD-10 Codes 'S04', 'S06', 'S07', 'S08', 'S17', 'S18', 'S01.7', 'S02.0', 'S02.1', 'S02.6', 'S02.7', 'S03.0', 'S09.0', 'S09.2', 'S09’, S05.2', 'S05.7' †Patients with unknown triage category (N=713 out of 17,700, 4.0%) were included in analyses.
