# Supplementary material for: The impact of disease severity adjustment on hospital standardised mortality ratios: Results from a service-wide analysis of ischaemic stroke admissions using linked pre-hospital, admissions and mortality data
Source: PLoS One. 2019 May 21;14(5):e0216325. doi: 10.1371/journal.pone.0216325 (PMC6528964; doi:10.1371/journal.pone.0216325)
Supplement: S2 Appendix — (DOCX) [file pone.0216325.s002.docx]

**S2 Appendix: Patient characteristics and 30-day mortality**

| **Variable** | **Alive (%)** | **Died (%)** | **Total N (%)*** |
| --- | --- | --- | --- |
| ***Sex*** |  |  |  |
| Male | 8,165 (88.5) | 1,063 (11.5) | 9,228 (52.1) |
| Female | 6,922 (81.7) | 1,550 (18.3) | 8,472 (47.9) |
|  |  |  |  |
| ***Index of Relative***  ***Socio-economic Disadvantage*** |  |  |  |
| Quartile 1 (Greatest) | 3,674 (84.7) | 662 (15.3) | 4,336 (24.5) |
| Quartile 2 | 3,000 (82.6) | 634 (17.4) | 3,634 (21.1) |
| Quartile 3 | 3,016 (85.0) | 532 (15.0) | 3,548 (20.0) |
| Quartile 4 | 2,672 (86.2) | 427 (13.8) | 3,099 (17.5) |
| Quartile 5 (Least) | 2,725 (88.4) | 358 (11.6) | 3,083 (17.4) |
|  |  |  |  |
| ***Previous stroke*** |  |  |  |
| No | 13,478 (85.9) | 2,213 (14.1) | 15,691 (88.6) |
| Yes | 1,609 (80.1) | 400 (19.9) | 2,009 (11.4) |
|  |  |  |  |
| ***Atrial fibrillation*** |  |  |  |
| No | 9,871 (88.6) | 1,269 (11.4) | 11,140 (62.9) |
| Yes | 5,216 (79.5) | 1,344 (20.5) | 6,560 (37.1) |
|  |  |  |  |
| ***Most urgent Triage category***  ***(“Life-threatening”)*** |  |  |  |
| No | 14,526 (87.5) | 2,078 (12.5) | 16,604 (93.8) |
| Yes | 561 (51.2) | 535 (48.8) | 1,096 (6.2) |
|  |  |  |  |
| **Total patients** | **15,087 (85.2)** | **2,613 (14.8)** | **17,700 (100)** |
| **Mean Age (SD)** | 74 (14) | 83 (11) | 75 (14) |
| **Median Age (25^th^-75^th^ percentiles** | 76 (65-84) | 85 (78-90) | 78 (67-85) |

*Percentage of total number of patients (N=17,700).

S2 Appendix continued. Charlson Comorbidities* and 30-day mortality (%)

| Comorbidity | Alive  N (% Row) | Dead  N (% Row) | Total  N/17,700 (%Total ) |
| --- | --- | --- | --- |
| Myocardial infarction | 1,909 (77.8) | 545 (22.2) | 2,454 (13.9%) |
| Congestive heart failure | 2,013 (70.5) | 841 (29.5) | 2,854 (16.1%) |
| Peripheral vascular disease | 1,318 (79.3) | 344 (20.7) | 1,662 (9.4%) |
| Dementia | 1,250 (67.6) | 599 (32.4) | 1,849 (10.4%) |
| Chromic pulmonary disease | 1,592 (78.3) | 440 (21.7) | 2,032 (11.5%) |
| Connective tissue disease | 305 (82.9) | 63 (17.1) | 368 (2.1%) |
| Peptic ulcer | 455 (81.0) | 107 (19.0) | 562 (3.2%) |
| Mild liver disease | 301 (83.8) | 58 (16.2) | 359 (2.0%) |
| Diabetes without chronic complications | 3,261 (85.5) | 552 (14.5) | 3,813 (21.5%) |
| Diabetes with chronic complications) | 2,882 (85.0) | 507 (15.0) | 3,389 (19.1%) |
| Renal disease | 1,708 (77.4) | 498 (22.6) | 2,206 (12.5%) |
| Non-metastatic malignancy | 1,599 (81.1) | 373 (18.9) | 1,972 (11.1%) |
| Moderate/severe liver disease | 80 (82.5) | 17 (17.5) | 97 (0.5%) |
| Metastatic malignancy | 387 (72.9) | 144 (27.1) | 531 (3.0%) |

*Adapted as per Goldstein et al^23^ for use in patients with stroke (removing cerebrovascular diseases and hemiplegia). ICD-10 Codes obtained from Quan et al^24^
