# Supplementary material for: The impact of disease severity adjustment on hospital standardised mortality ratios: Results from a service-wide analysis of ischaemic stroke admissions using linked pre-hospital, admissions and mortality data
Source: PLoS One. 2019 May 21;14(5):e0216325. doi: 10.1371/journal.pone.0216325 (PMC6528964; doi:10.1371/journal.pone.0216325)
Supplement: S3 Appendix — (PDF) [file pone.0216325.s003.pdf]

S3 Appendix: Cross-validation of risk-adjusted models.*

Model	Akaike Information Criterion	Nagelkerke r^2	c-statistic 95% CI	Brier Score
Standard Models				
Model 1: Base+Comorbidities**	13,309	0.17	0.75 (0.74-0.76)	0.12
Model 2: Base+Comorbidities+Stroke Severity	11,580	0.32	0.83 (0.82-0.84)	0.10
Enhanced models				
Model 1: Base+Comorbidities+Arrival mode†+Most urgent ED triage category	12,482	0.24	0.79 (0.78-0.80)	0.11
Model 2: Base+Comorbidities+Stroke severity+Most urgent ED triage category‡	11,494	0.32	0.83 (0.82-0.84)	0.10

*Models were cross-validated with an external data-set applying the same selection criteria used to derive data for the main study period. The external data-set comprised three years of linked administrative data recording information about 17,339 ischaemic stroke patients from July 1, 2008 to June 30, 2011. **Base variables are: Age, age*age, sex, year of admission, prior stroke and a measure of socio-economic status based on patient location of residence. Comorbidities include Charlson comorbidities+Atrial Fibrillation; †Ambulance or private transport; ‡Note: Stroke severity measure for Model 2 incorporates arrival mode.
