# Supplementary material for: The impact of disease severity adjustment on hospital standardised mortality ratios: Results from a service-wide analysis of ischaemic stroke admissions using linked pre-hospital, admissions and mortality data
Source: PLoS One. 2019 May 21;14(5):e0216325. doi: 10.1371/journal.pone.0216325 (PMC6528964; doi:10.1371/journal.pone.0216325)
Supplement: S4 Appendix — (PDF) [file pone.0216325.s004.pdf]

S4 Appendix: Change in outlier status of comorbidity adjusted HSMRs with and without stroke severity adjustment (Enhanced models)

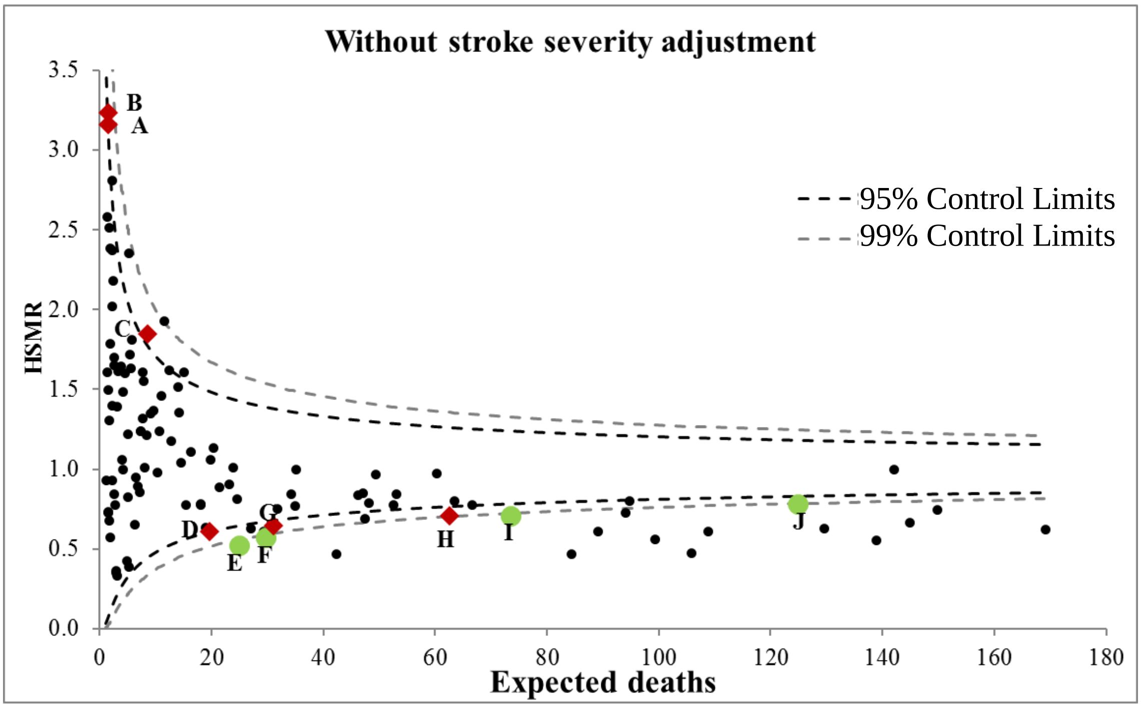

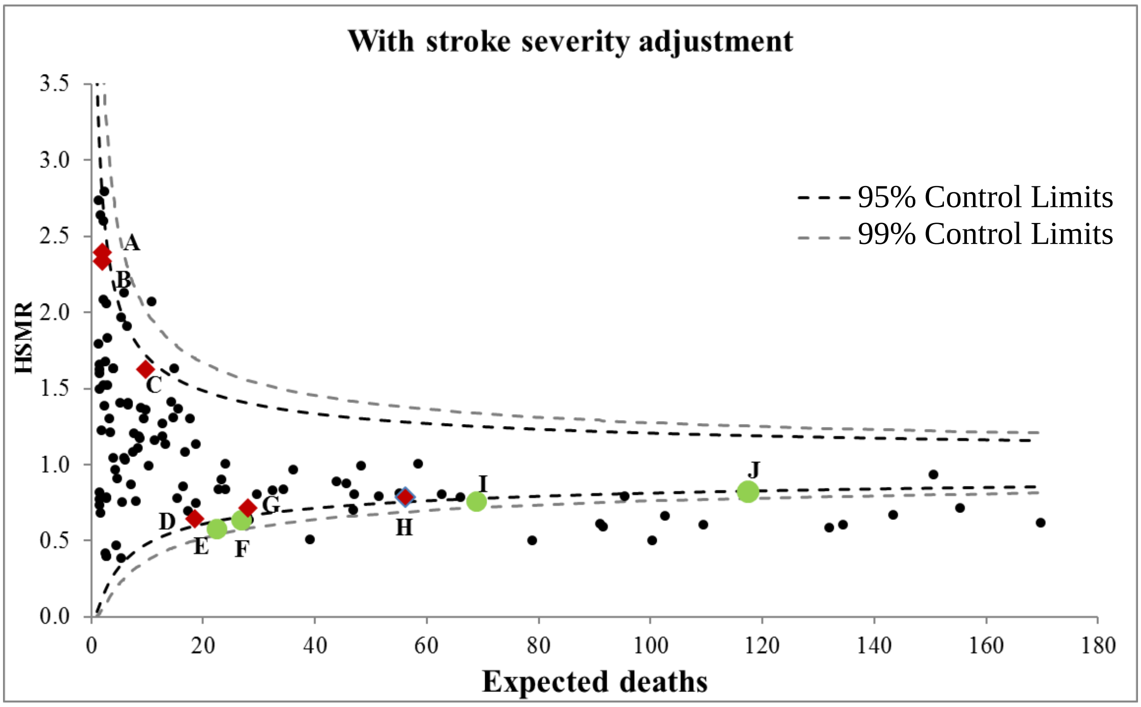

Legend

◆ Stroke severity adjustment changes health service from an outlier to a non-outlier

● Alert signal "downgraded" from an 99% to 95% control limit outlier with stroke severity adjustment.
