# Supplementary material for: The impact of disease severity adjustment on hospital standardised mortality ratios: Results from a service-wide analysis of ischaemic stroke admissions using linked pre-hospital, admissions and mortality data
Source: PLoS One. 2019 May 21;14(5):e0216325. doi: 10.1371/journal.pone.0216325 (PMC6528964; doi:10.1371/journal.pone.0216325)
Supplement: S5 Appendix — (DOCX) [file pone.0216325.s005.docx]

**S5 Appendix: Results of sensitivity analysis including cases with presumed “misclassified stroke” defined as cases with stroke diagnosis revised upon early transfer to another hospital and those discharged home alive within 48 hours of presentation.**

Table 1: Goodness of fit for 30-day mortality: Main analysis (N=17,700) versus sensitivity analysis including misclassified stroke and discharged alive within 48 hours (N=19,211)

| **Model*** | **AIC** | | **Nagelkerke r^2^** | | **AUC** | | **Brier Score** | |
| --- | --- | --- | --- | --- | --- | --- | --- | --- |
| *Analysis* | *Main* | *Sensitivity* | *Main* | *Sensitivity* | *Main* | *Sensitivity* | *Main* | *Sensitivity* |
| Comorbidity adjusted (Standard) | 13,048 | 13,603 | 0.17 | 0.18 | 0.75 | 0.76 | 0.11 | 0.11 |
| Comorbidity and severity adjusted (Standard) | 11,487 | 11,957 | 0.31 | 0.31 | 0.82 | 0.83 | 0.10 | 0.09 |
| Comorbidity adjusted (Enhanced) | 12,145 | 12,625 | 0.25 | 0.26 | 0.80 | 0.81 | 0.11 | 0.10 |
| Comorbidity and severity adjusted (Enhanced) | 11,334 | 11,790 | 0.32 | 0.33 | 0.83 | 0.84 | 0.10 | 0.09 |

**Table 2: Correlation of HSMRs derived from models which either include or exclude patients with suspected misclassification (that is, those cases with revised diagnosis upon early transfer) and early discharged cases to home (within 48 hours).**

| **Model*** | **Spearman’s Rho** | **95% CI** |
| --- | --- | --- |
| Comorbidity adjusted (Standard) | 0.98 | 0.97 to 0.99 |
| Comorbidity and severity adjusted (Standard) | 0.95 | 0.93 to 0.97 |
| Comorbidity adjusted (Enhanced) | 0.97 | 0.96 to 0.98 |
| Comorbidity and severity adjusted (Enhanced) | 0.96 | 0.94 to 0.97 |

Table 3: Number of outliers and inliers using comorbidity adjusted (Model 1) funnel plot results compared against “gold standard” severity and comorbidity adjusted (Model 2) funnel plot results (Standard Models)

|  | **Gold Standard Risk Adjustment (Model 2)**  **N=117 Hospitals** | | | | |
| --- | --- | --- | --- | --- | --- |
|  | **95% Control Limit** | | **99% Control Limit** | | |
|  | Outliers | Inliers | | Outliers | Inlier |
| **Comorbidity Adjustment (Model 1)** |  |  | |  |  |
| Outlier | 15 | 7 | | 10 | 1 |
| Inlier | 4 | 91 | | 2 | 104 |

| **Accuracy metric** | **95% Control Limits** | **99% Control Limits** |
| --- | --- | --- |
| Percent agreement* | (91+15)/117=91% | (104+10)/117=97% |
| Kappa (95% CI) | 0.68 (0.50-0.85) | 0.86 (0.70-1.00) |
| Sensitivity (95% CI)† | 15/(15+4)=79% (54%-94%) | 10/(10+2)=83% (52%-98%) |
| PPV (95% CI) †† | 15/(15+7)=68% (50%-82%) | 10/(10+1)=91% (58%-99%) |
| False Positive Rate‡ | 7/(7+91)=7% | 1/(1+104)=<1% |
| False Negative Rate‡‡ | 4/(4+15)=21% | 2/(2+10)=17% |

*Percentage of hospitals with concordant classifications between comorbidity (Model 1) and gold-standard adjustment (that is, comorbidity and severity adjustment, Model 2).

†Proportion of true outliers according to gold-standard comorbidity and severity risk adjustment (Model 2) detected as outliers according comorbidity adjustment alone (Model 1).

††Proportion of outliers detected by comorbidity risk adjusted modelling (Model 1) that are true outliers according to gold-standard severity and comorbidity adjustment (Model 2).

‡Number of hospitals falsely detected as outliers using comorbidity risk adjustment alone (Model 1) divided by the number of “inlier” hospitals according to gold-standard risk adjustment (Model 2).

‡‡Number of hospitals missed as “true outliers” using comorbidity risk adjustment alone (Model 1) divided by the number of “true outlier” hospitals according to gold-standard risk adjustment (Model 2)

Table 4: Number of outliers and inliers using comorbidity adjusted (Model 1) funnel plot results compared against “gold standard” severity and comorbidity adjusted (Model 2) funnel plot results (Enhanced Models)

|  | **Gold Standard Risk Adjustment (Model 2)**  **N=117 Hospitals** | | | | |
| --- | --- | --- | --- | --- | --- |
|  | **95% Control Limit** | | **99% Control Limit** | | |
|  | Outliers | Inliers | | Outliers | Inlier |
| **Comorbidity Adjustment (Model 1)** |  |  | |  |  |
| Outlier | 19 | 6 | | 13 | 2 |
| Inlier | 0 | 92 | | 1 | 101 |

| **Accuracy metric** | **95% Control Limits** | **99% Control Limits** |
| --- | --- | --- |
| Percent agreement* | (92+19)/117=95% | (101+13)/117=97% |
| Kappa (95% CI) | 0.83 (0.70-0.96) | 0.88 (0.75-1.00) |
| Sensitivity (95% CI)† | 19/(19+0)=100% (82%-100%) | 13/(13+1)=93% (66%-100%) |
| PPV (95% CI) †† | 19/(19+6)=76% (59%-87%) | 13/(13+2)=87% (62%-97%) |
| False Positive Rate‡ | 6/(6+92)=6% | 2/(2+101)=2% |
| False Negative Rate‡‡ | 0/(0+19)=0% | 1/(1+13)=7% |

*Percentage of hospitals with concordant classifications between comorbidity (Model 1) and gold-standard adjustment (that is, comorbidity and severity adjustment, Model 2).

†Proportion of true outliers according to gold-standard comorbidity and severity risk adjustment (Model 2) detected as outliers according comorbidity adjustment alone (Model 1).

††Proportion of outliers detected by comorbidity risk adjusted modelling (Model 1) that are true outliers according to gold-standard severity and comorbidity adjustment (Model 2).

‡Number of hospitals falsely detected as outliers using comorbidity risk adjustment alone (Model 1) divided by the number of “inlier” hospitals according to gold-standard risk adjustment (Model 2).

‡‡Number of hospitals missed as “true outliers” using comorbidity risk adjustment alone (Model 1) divided by the number of “true outlier” hospitals according to gold-standard risk adjustment (Model 2)

Figure 1: Bland Altman plots: Comparison of differences in rank order or RAMRs (Standard model)

Figure 2: Bland Altman plots: Comparison of differences in rank order or RAMRs (Enhanced model)

Table 5: Summary of differences in rank order results between main and sensitivity analyses.

| **Rank order results** | **Standard Models** | | **Enhanced Models** | |
| --- | --- | --- | --- | --- |
| **Analysis** | ***Sensitivity Analysis*** | ***Main Analysis*** | ***Sensitivity Analysis*** | ***Main Analysis*** |
| ***Range*** | 0-28 places | 0-22 | 0-16 | 0-17 |
| ***Median difference*** | 4 | 4 | 3 | 3 |
| ***Inter-quartile Range*** | 1.5 to 6 | 2 to 7 | 1 to 6 | 1 to 5 |
| ***Dispersion of rank differences*** | ± 13.9 places | ±13.8 | ±10.1 | ±9.8 |

Table 6: Comparison of results between main analysis and sensitivity analysis: outlier classification using funnel plots

| **Metric** | **Standard Models** | | | | **Enhanced Models** | | | |
| --- | --- | --- | --- | --- | --- | --- | --- | --- |
| **Adjustment** | **95% Control Limits** | | **99% Control Limits** | | **95% Control Limits** | | **99% Control Limits** | |
| **Analysis** | ***Sensitivity Analysis*** | ***Main Analysis*** | ***Sensitivity Analysis*** | ***Main Analysis*** | ***Sensitivity Analysis*** | ***Main Analysis*** | ***Sensitivity Analysis*** | ***Main Analysis*** |
| ***Agreement*** | 91% | 89% | 97% | 97% | 95% | 95% | 97% | 96% |
| ***Kappa*** | 0.68 | 0.67 | 0.86 | 0.85 | 0.83 | 0.85 | 0.88 | 0.85 |
| ***Sensitivity*** | 79% | 74% | 83% | 83% | 100% | 100% | 93% | 100% |
| ***PPV*** | 68% | 74% | 91% | 91% | 76% | 80% | 87% | 76% |
| ***FP*** | 7% | 7% | <1% | 1% | 6% | 7% | 2% | 4% |
| ***FN*** | 21% | 26% | 17% | 17% | 0% | 0% | 7% | 0% |
